# Prognosis and Chemotherapeutic Efficacy in Extrahepatic Cholangiocarcinoma With Lung Metastases

**DOI:** 10.1002/cnr2.70236

**Published:** 2025-05-24

**Authors:** Chao Zhang, Shun Tu, Yanting Liao, Yaqiang Shu, Muyu Fu, Jiayue Li, Xiaohua Lei

**Affiliations:** ^1^ The First Affiliated Hospital, Department of Hepatobiliary Surgery Hengyang Medical School, University of South China Hengyang Hunan China; ^2^ Department of Public Health Leifeng Street Community Health Service Centre Changsha Hunan China

**Keywords:** chemotherapy, extrahepatic cholangiocarcinoma, lung metastasis, prognosis, SEER

## Abstract

**Objective:**

Studies on lung metastases from extrahepatic cholangiocarcinoma (ECC) are rare. This study aims to fill this gap by analyzing the influencing factors, prognosis, and chemotherapeutic efficacy of ECC lung metastases, and to provide insights for optimizing medical care for patients with ECC lung metastases.

**Methods:**

We retrieved data from the Surveillance, Epidemiology and End Results (SEER) database for patients with metastatic ECC (stage M1) from 2018 to 2021. The study analyzed these characteristics using descriptive statistics. To calculate Hazard Ratios (HR), multivariate COX regression analyses were performed. Overall survival (OS) was estimated using the Kaplan–Meier method, and the survival of patients between groups was compared using the log‐rank test.

**Results:**

A total of 762 people participated in the study, 50.4% of whom were men. At the time of diagnosis, 17.8% of patients had pulmonary metastases. 52.5% received chemotherapy. Multivariate COX analysis identified lung metastases as a significant risk factor for death from metastatic ECC (HR 1.64, CI 1.32–2.03, *p* < 0.001). Treatment with chemotherapy (HR 0.20, CI 0.17–0.25, *p* < 0.001) and female sex (HR 0.80, CI 0.67–0.94, *p* = 0.008) were associated with a better prognosis. Therefore, we further compared the prognosis and chemotherapy outcomes of male and female patients with ECC lung metastases. The median survival of male patients with and without lung metastases was 2 and 5 months, respectively (*p* = 0.016), whereas there was no significant difference in female patients (*p* = 0.19). Regardless of gender, patients with lung metastases had significantly worse OS even after receiving chemotherapy (*p* = 0.0065 in the male group and *p* = 0.0075 in the female group). Regardless of gender, patients with lung metastases who did not receive chemotherapy had significantly shorter overall survival than those who received chemotherapy. Not receiving chemotherapy vs. receiving chemotherapy (male: 1 month vs. 5 months, *p* < 0.0001; female: 2 months vs. 9 months, *p* < 0.0001).

**Conclusion:**

Pulmonary metastasis is an important prognostic factor in ECC and is associated with poorer survival, especially in male patients. Therefore, preventive measures and effective control of lung metastases (e.g., chemotherapy), especially in male patients, may improve survival in patients with ECC.

## 1 Introduction

The bile duct epithelium is the source of cholangiocarcinoma (CCA), which is a highly lethal tumor. Anatomically, it can be divided into extrahepatic cholangiocarcinoma (ECC) and intrahepatic cholangiocarcinoma [1]. ECC accounts for approximately 70%–90% of cholangiocarcinomas and is the most common primary biliary tract cancer [2]. The global incidence of ECC has been consistently higher than 0.001% between 2001 and 2016 [1, 3], and the 5‐year survival rate for metastatic ECC is only 2% [4]. For non‐metastatic ECC, surgery is the best treatment option [5]. However, due to poor diagnostic methods, some patients are diagnosed with advanced disease with metastasis and thus lose the best time for surgery.

Liver and lymph nodes are frequently affected by metastasis in ECC, whereas lung metastasis is uncommon. However, literature indicates that patients with pulmonary metastasis are more likely to have metastases in other organs [6]. Currently, there are very few studies on pulmonary metastasis in ECC, so there is a lack of comprehensive understanding of its incidence and prognostic significance. Current guidelines generally consider ECC with distant metastases as a contraindication to surgery [5]. Therefore, other therapeutic options that include chemotherapy are of particular importance.

According to the most recent clinical guidelines, the first‐line treatment for advanced or metastatic ECC is cisplatin plus gemcitabine chemotherapy in combination with the anti‐PD‐L1 inhibitor durvalumab. Early molecular profiling of systemic tumors is recommended, and if applicable, molecularly targeted therapies such as FGFR2 or IDH‐targeted agents may be used. If not applicable, FOLFOX chemotherapy is the only second‐line standard of care. To date, no third‐line chemotherapy standard has been validated [7]. However, to the best of our knowledge, the major trials identifying first‐line treatments have not analyzed lung metastases as a separate subgroup. Metastatic disease is often difficult to cure because systemic and disseminated tumor cells become resistant to existing therapies [8]. Thus, the efficacy of chemotherapy for ECC with lung metastases is unclear. To address this research gap, we conducted an in‐depth study of all patients with metastatic (including lung metastases) ECC using the Surveillance, Epidemiology, and End Results (SEER) database to understand the independent influences on metastatic ECC. In addition, the study sought to assess the impact of lung metastases on survival outcomes in patients with ECC, as well as the potential clinical benefit of using chemotherapy in ECC patients with lung metastases. The results of the study will provide insights to optimize medical care for patients with ECC lung metastases as well as suggest new areas of research for future randomized clinical trials.

## 2 Methods

### 2.1 Data

Data for this research was obtained from 17 population‐based registries within the SEER database of the US National Cancer Institute covering the years 2018 to 2021 (submitted in November 2023). The SEER‐17 project represents approximately 26.5% of the US population. The extensive size of the SEER database allows for more accurate extrapolation of results to the general population compared to studies conducted at a single center.

### 2.2 Information Selection

Since the 8th edition of the American Joint Committee on Cancer (AJCC) staging system was released and adopted in 2018, patient data from 2018 to 2021 were selected for this study. First, we included patients with International Classification of Diseases for Oncology (ICD‐O‐3:24) topographic codes retrieving malignancies with a primary site in the extrahepatic bile ducts; second, all patients were those with a TNM stage of M1 in the AJCC (8th edition) staging subgroup IV. Finally, patients with missing variable information were excluded, and all patients had complete survival, metastasis, and chemotherapy information.

The endpoints of analysis were survival status and overall survival (OS).

### 2.3 Research Variables

The following variables were selected from the SEER: Age at diagnosis, Sex, Race (categorized as White, Black, and Other (including American Indian, Alaska Native, Asian or Pacific Islander)), Marital status (categorized as Married (including Cohabiting) and Unmarried (including Divorced, Separated, Single, Unmarried or Domestic Partner, and Widowed)), Pathology types (categorized as Adenocarcinoma (8140/3: Adenocarcinoma, NOS), Cholangiocarcinoma (8160/3: Cholangiocarcinoma), and Other (wanting to include all cancers arising from the bile ducts, regardless of pathological subtype, including 8000/3: Neoplasm, malignant; 8010/3: Carcinoma, NOS; 8013/3: Large cell neuroendocrine carcinoma; 8020/3: Carcinoma, undifferentiated, NOS; 8041/3: Small cell carcinoma, NOS; 8070/3: Squamous cell carcinoma, NOS; 8162/3: Klatskin tumor; 8246/3: Neuroendocrine carcinoma, NOS; 8480/3: Mucinous adenocarcinoma; 8490/3: Signet ring cell carcinoma; 8560/3: Adenosquamous carcinoma)), Chemotherapy, Sites of distant metastasis, Survival status, and Survival time. Patients were categorized into two groups based on the presence or absence of pulmonary metastasis to assess its prognostic significance. Additionally, patients were stratified based on whether they received chemotherapy to analyze its treatment effects specifically in those with lung metastasis. The study included all metastatic ECC patients reported in the SEER database from 2018 to 2021, with the primary endpoint being death from any cause. Survival time was defined as the duration from the date of diagnosis to the date of death or the last follow‐up in 2021 for surviving patients. These parameters were utilized to comprehensively analyze survival outcomes and chemotherapy impacts within the specified study period.

### 2.4 Statistical Analysis

Data analysis was performed using R software version 4.3.3. Demographic and clinical characterization was carried out using descriptive statistics. A multifactorial COX proportional risk regression model was utilized to conduct analyses in order to calculate hazard ratios (HR) and 95% confidence intervals (CI). Overall survival rate was estimated using the Kaplan–Meier technique, with survival rates between groups compared using the log‐rank test. Statistical significance was determined for each analysis method if *p* < 0.05.

## 3 Results

### 3.1 Patient Information Characteristics

**FIGURE 1:**
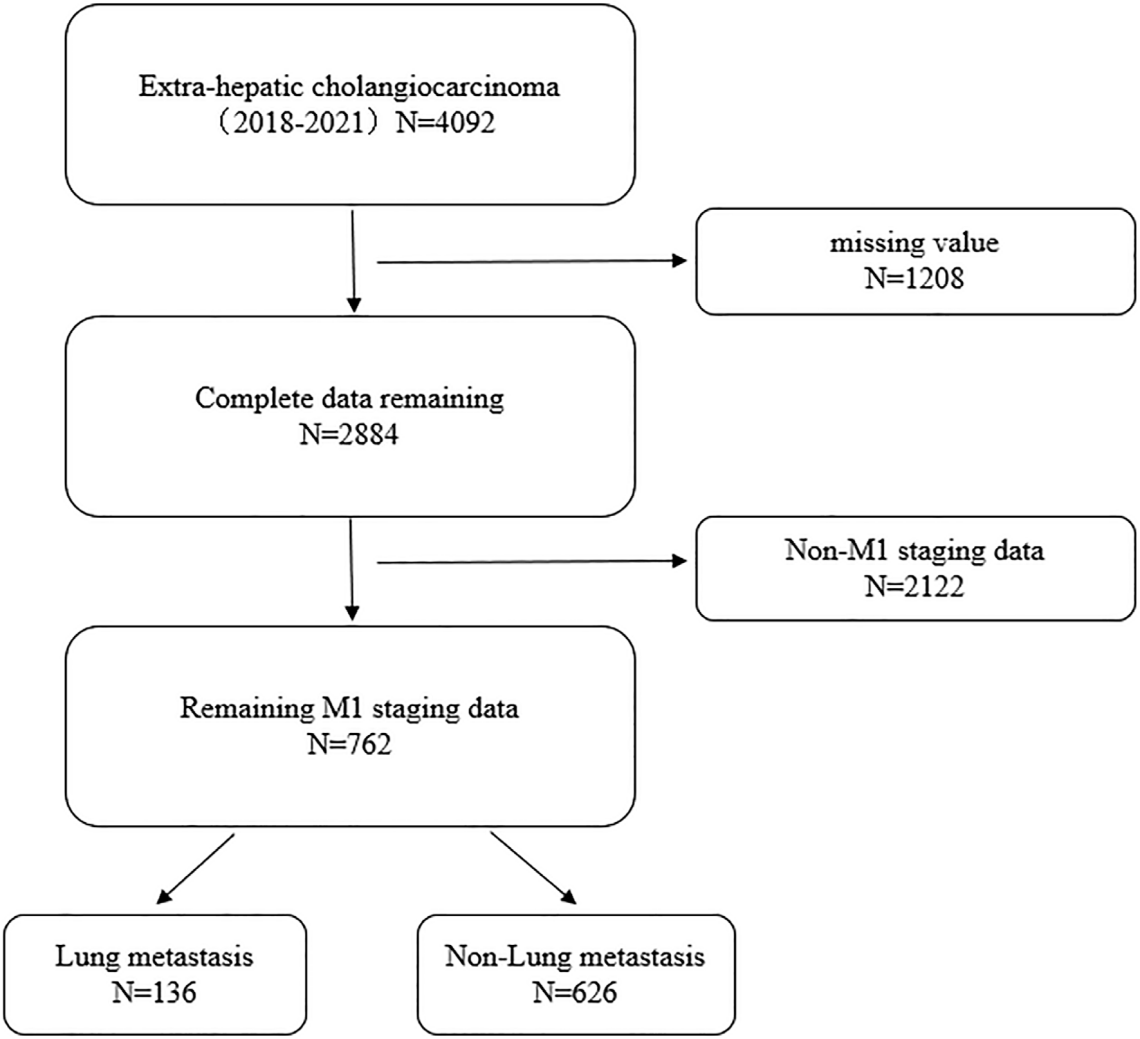
Patient selection flowchart.

762 patients were included in the study analysis and the flow is shown in Figure 1. According to World Health Organization (WHO) classification, individuals aged 60 years and above are considered elderly; hence, the age variable in this study was grouped into < 60 and ≥ 60 years, with a range from 25 to 85+. In this research, 50.4% (*n* = 384) of the patients were male and 49.6% (*n* = 378) were female. A total of 75.1% (*n* = 572) of the patients were White, 10% (*n* = 76) were Black, and 15% (*n* = 114) belonged to other races. 52.4% (*n* = 399) of the patients were married, and 47.6% (*n* = 363) were unmarried. 54.6% (*n* = 416) had adenocarcinoma, 36.5% (*n* = 278) had cholangiocarcinoma, and 8.9% (*n* = 68) had other pathological types. In this study, 52.5% (*n* = 400) of metastatic ECC patients received chemotherapy. The distribution of metastatic sites was as follows: 17.8% (*n* = 136) to the lungs, 9.2% (*n* = 70) to the bones, 0.9% (*n* = 7) to the brain, and 63.4% (*n* = 483) to the liver. As of the last follow‐up in 2021, 23.6% (*n* = 180) of patients were still alive, whereas 76.4% (*n* = 582) had died, with 3 months being the total median survival time. These characteristics have been listed in Table 1.

**TABLE 1:** Baseline characteristics of patients with metastatic extrahepatic cholangiocarcinoma classified according to receipt of chemotherapy.

Variable	Overall, *n* = 762	Chemotherapy treatment	
		No, *n* = 362	Yes, *n* = 400
Age (year)			
> 60	153 (20.1%)	41 (11.3%)	112 (28.0%)
≥ 60	609 (79.9%)	321 (88.7%)	288 (72.0%)
Sex			
Male	384 (50.4%)	177 (48.9%)	207 (51.7%)
Female	378 (49.6%)	185 (51.1%)	193 (48.3%)
Race			
White	572 (75.1%)	275 (76.0%)	297 (74.3%)
Black	76 (10.0%)	31 (8.6%)	45 (11.2%)
Other	114 (15.0%)	56 (15.4%)	58 (14.5%)
Marital status			
Married	399 (52.4%)	160 (44.2%)	239 (59.8%)
Unmarried	363 (47.6%)	202 (55.8%)	161 (40.2%)
Histology			
Adenocarcinoma	416 (54.6%)	200 (55.2%)	216 (54.0%)
Cholangiocarcinoma	278 (36.5%)	127 (35.1%)	151 (37.8%)
Other	68 (8.9%)	35 (9.7%)	33 (8.2%)
Lung metastasis	136 (17.8%)	55 (15.2%)	81 (20.3%)
Bone metastasis	70 (9.2%)	31 (8.6%)	39 (9.8%)
Brain metastasis	7 (0.9%)	4 (1.1%)	3 (0.8%)
Liver metastasis	483 (63.4%)	241 (66.6%)	242 (60.5%)
Vital status			
Alive	180 (23.6%)	41 (11.3%)	139 (34.8%)
Dead	582 (76.4%)	321 (88.7%)	261 (65.2%)
Survival months			
Median (IQR)	3 (1, 8)	1 (0, 3)	7 (3, 12)
Range (min, max)	0, 43	0, 35	0, 43

*Note:* Data are *n* (%) unless otherwise specified.

Abbreviation: IQR, interquartile range.

17.8% (*n* = 136) of the patients had lung metastases at diagnosis, with or without other site metastases. 13.2% (*n* = 18), 1.5% (*n* = 2), 57.4% (*n* = 78) of patients with ECC with lung metastases had concurrent bone, brain, and liver metastases at diagnosis. The elderly accounted for 77.2% (*n* = 105). 52.9% (*n* = 72) of the patients were male, and 47.1% (*n* = 64) were female. The majority were White, 75.7% (*n* = 103), with smaller proportions being Black, 11.1% (*n* = 15), and other races, 13.2% (*n* = 18). 50.7% (*n* = 69) of the patients were married, and 49.3% (*n* = 67) were unmarried. 58.1% (*n* = 79) had adenocarcinoma, 32.4% (*n* = 44) had cholangiocarcinoma, and 9.5% (*n* = 13) had other pathological types. A total of 59.6% (*n* = 81) of the patients received chemotherapy. As at the end of the last follow‐up, 20.6% (*n* = 28) of patients were still alive, whereas 79.4% (*n* = 108) had died, with 2 months being the total median survival time. Table 2 lists the baseline characteristics of these patients.

**TABLE 2:** Baseline characteristics of patients with metastatic extrahepatic cholangiocarcinoma classified according to the presence or absence of lung metastases.

Variable	Overall, *n* = 762	Lung metastasis	
		No, *n* = 626	Yes, *n* = 136
Age (year)			
> 60	153 (20.1%)	122 (19.5%)	31 (22.8%)
≥ 60	609 (79.9%)	504 (80.5%)	105 (77.2%)
Sex			
Male	384 (50.4%)	312 (49.8%)	72 (52.9%)
Female	378 (49.6%)	314 (50.2%)	64 (47.1%)
Race			
White	572 (75.1%)	469 (74.9%)	103 (75.7%)
Black	76 (10.0%)	61 (9.8%)	15 (11.1%)
Other	114 (15.0%)	96 (15.3%)	18 (13.2%)
Marital status			
Married	399 (52.4%)	330 (52.7%)	69 (50.7%)
Unmarried	363 (47.6%)	296 (47.3%)	67 (49.3%)
Histology			
Adenocarcinoma	416 (54.6%)	337 (53.8%)	79 (58.1%)
Cholangiocarcinoma	278 (36.5%)	234 (37.4%)	44 (32.4%)
Other	68 (8.9%)	55 (8.8%)	13 (9.5%)
Chemotherapy	400 (52.5%)	319 (51.0%)	81 (59.6%)
Bone metastasis	70 (9.2%)	52 (8.3%)	18 (13.2%)
Brain metastasis	7 (0.9%)	5 (0.8%)	2 (1.5%)
Liver metastasis	483 (63.4%)	405 (64.7%)	78 (57.4%)
Vital status			
Alive	180 (23.6%)	152 (24.3%)	28 (20.6%)
Dead	582 (76.4%)	474 (75.7%)	108 (79.4%)
Survival months			
Median (IQR)	3 (1, 8)	3 (1, 9)	2 (1, 5)
Range (min, max)	0, 43	0, 43	0, 33

*Note:* Data are *n* (%) unless otherwise specified.

Abbreviation: IQR, interquartile range.

### 3.2 Factors Affecting Patient Survival

**FIGURE 2:**
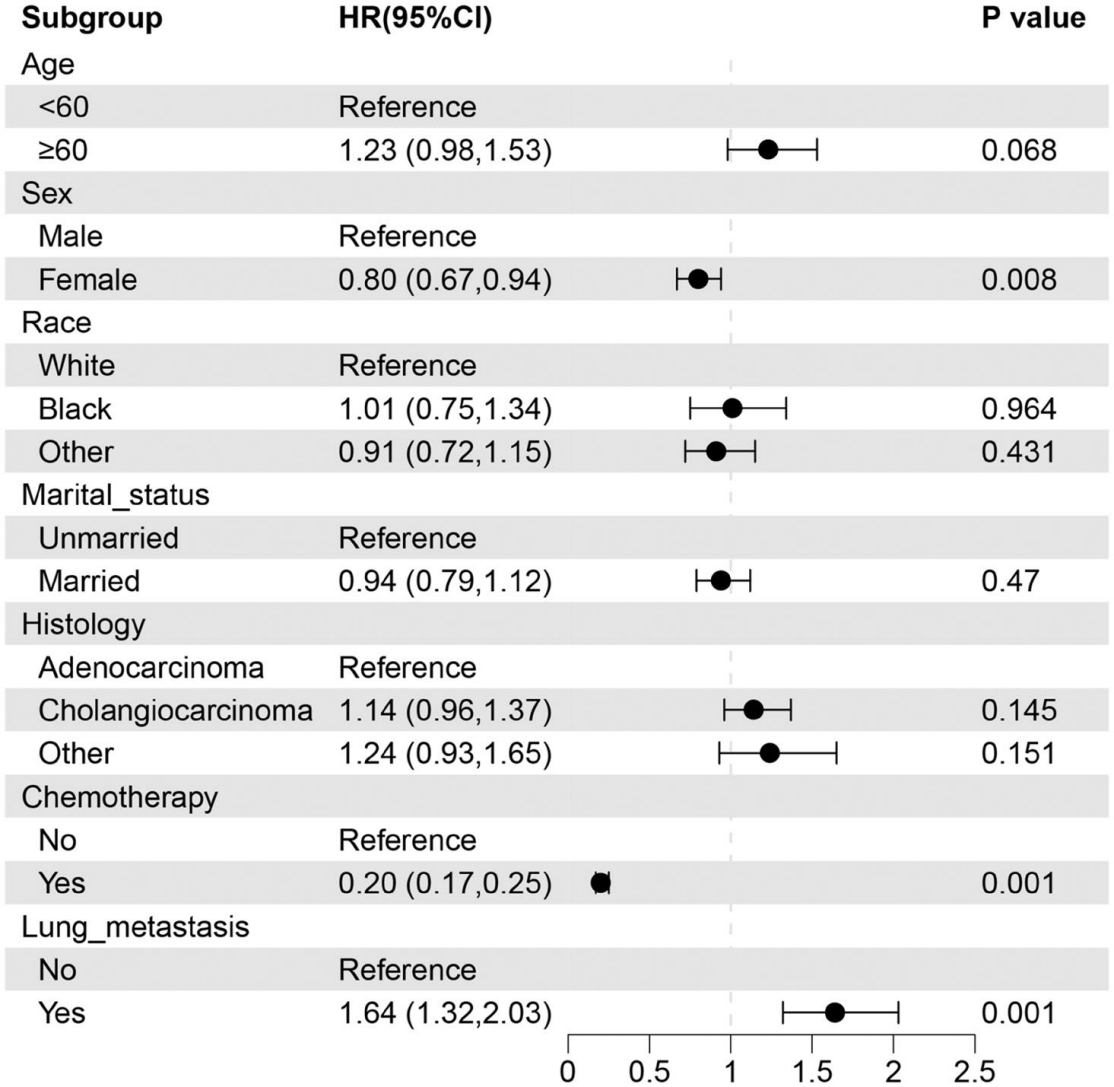
Hazard ratio using Cox proportional‐hazards analysis. Hazard ratios > 1 indicate an increased risk of mortality, while hazard ratios < 1 indicate a decreased risk of mortality. The *p*‐value for each variable is depicted on the right side of the figure. CI, confidence intervals; HR, hazard ratios.

Multifactorial COX proportional hazards analysis identified pulmonary metastases as a risk factor for patients with metastatic ECC, jeopardizing patient survival (HR 1.64, CI 1.32–2.03, *p* < 0.001). Improved OS was linked to being female (HR 0.80, CI 0.67–0.94, *p* = 0.008) and receiving chemotherapy (HR 0.20, CI 0.17–0.25, *p* < 0.001) (Figure 2). The main objective of this research was to investigate the prognosis of ECC lung metastases and the effect of chemotherapy; therefore, to minimize the effect of gender confounding, the patients were further analyzed by dividing them into two groups according to gender.

### 3.3 Prognostic Effects

**FIGURE 3:**
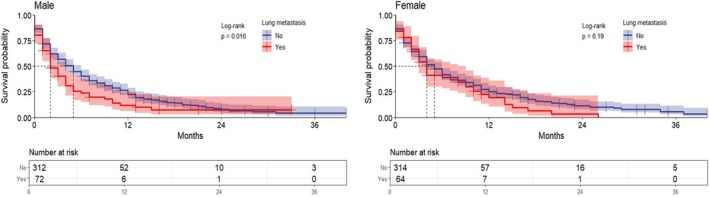
Overall survival in patients with metastatic extrahepatic cholangiocarcinoma based on lung metastases; the graphs represent the overall survival of the patients, and the risk tables represent the number of people at risk at the characteristic time points.

Separate one‐way survival analyses for sex‐stratified groups (men and women). The median OS for all metastatic ECC patients was 3 months. In the male group, the median OS for patients with lung metastases was 2 months compared to 5 months for those without lung metastases, indicating significantly lower OS in patients with lung metastases (*p* = 0.016). In contrast, the median OS of patients with and without lung metastases in the female group was 4 and 5 months, respectively, with no significant difference (*p* = 0.19) (Figure 3).

**FIGURE 4:**
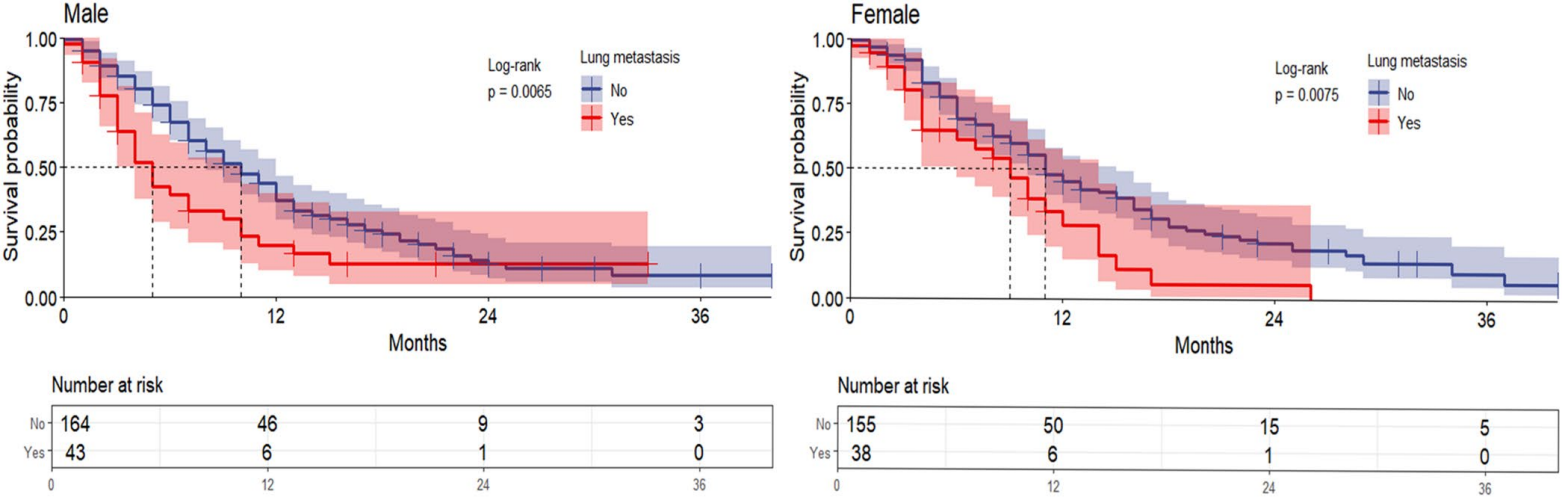
Overall survival of metastatic extrahepatic cholangiocarcinoma patients receiving chemotherapy for lung metastases; the graph represents overall patient survival and the risk table represents the number of people at risk at characteristic time points.

In the chemotherapy group, male ECC patients with lung metastases had a significantly lower OS than male ECC patients without lung metastases (median OS 5 months versus 10 months, *p* = 0.0065). Similarly, in the female group, patients without lung metastases had higher OS, with median OS of 9 months for those with lung metastases and 11 months for those without (*p* = 0.0075) (Figure 4).

**FIGURE 5:**
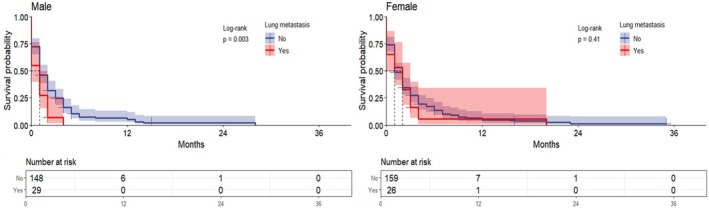
Overall survival in patients with metastatic extrahepatic cholangiocarcinoma who did not receive chemotherapy because of lung metastases; the graphs represent the overall survival of the patients, and the risk tables represent the number of people at risk at the characteristic time points.

In the non‐chemotherapy group, median survival was 1 month for both male ECC patients with and without lung metastases, but OS was significantly worse and statistically significant for those with lung metastases (*p* = 0.003). In contrast, OS was not statistically significant in the female group with or without lung metastases (*p* = 0.41) (Figure 5).

### 3.4 Effects of Chemotherapy

**FIGURE 6:**
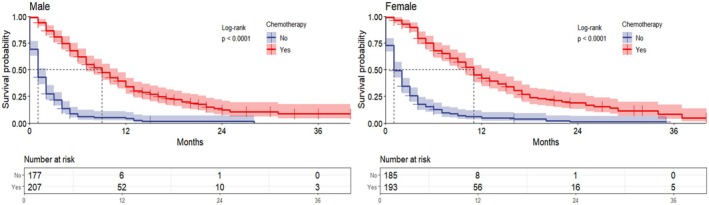
Overall survival in patients with metastatic extrahepatic cholangiocarcinoma treated with chemotherapy; the graph indicates overall patient survival and the risk table indicates the number of people at risk at characteristic time points.

**FIGURE 7:**
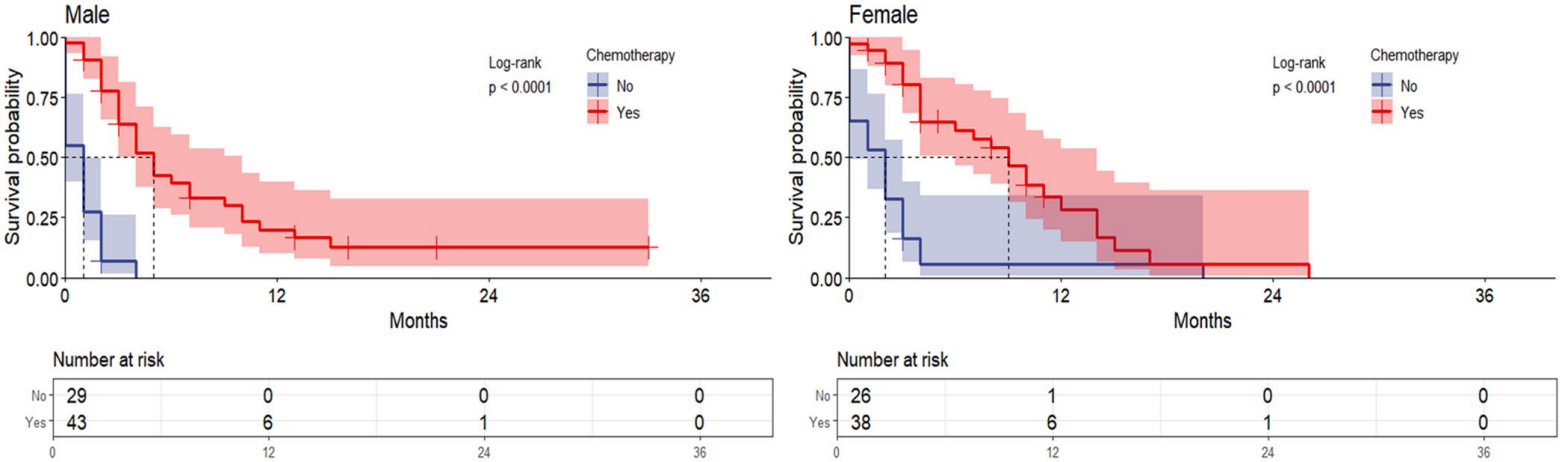
Overall survival based on the incidence of lung metastases in patients treated with chemotherapy for extrahepatic cholangiocarcinoma; the graphs represent the overall survival of the patients, and the risk tables represent the number of people at risk at the characteristic time points.

In all metastatic ECC, the median survival was 9 months and 1 month for male patients with and without chemotherapy, respectively (*p* < 0.0001), and 11 months and 1 month for females (*p* < 0.0001) (Figure 6). In ECC with lung metastases, the median survival was 5 months and 1 month for male patients with and without chemotherapy, respectively (*p* < 0.0001). While in female patients, it was 9 and 2 months (*p* < 0.0001), respectively (Figure 7). This demonstrated a statistically significant prolongation of OS in patients who received chemotherapy compared to patients with metastatic ECC who did not receive chemotherapy.

## 4 Discussion

CCA is characterized by insidious onset, lack of early symptoms, high invasiveness, and lack of effective screening, leading to rapid disease progression and making prevention difficult, posing a significant threat to human health. Current research on CCA mostly focuses on intrahepatic cholangiocarcinoma, with relatively less research on ECC. Clinically, ultrasound, CT, and MRI are commonly used for the preliminary diagnosis of ECC [4, 9], and the definitive diagnosis of ECC requires obtaining tissue from the lesion site via endoscopic ultrasound for pathological biopsy [10]. In the treatment of ECC, there are options such as radical surgical resection, radiotherapy, chemotherapy, photodynamic therapy, targeted therapy, and immunotherapy. Current guidelines consider surgery as the best curative option for resectable patients [5]. Unfortunately, certain individuals with ECC are already in the late stages with metastases in other organs when diagnosed, resulting in the inability to undergo surgery. Therefore, other treatments (e.g., chemotherapy) become critical. Studies have shown that distant metastasis is negatively correlated with patient survival time, leading to poorer prognosis, and ECC patients with lung metastasis are more likely to have other organ involvement [6]. Therefore, ECC with lung metastases is very valuable to study. Currently, reports on ECC lung metastasis are limited, and more research is needed to provide further clinical reference. Our study evaluated the outcome of metastatic ECC patients with concurrent lung metastases using the SEER database to address this gap. This is the largest study to date on factors influencing ECC lung metastases and the use of chemotherapy after lung metastases. We found a positive correlation between lung metastases and low OS in ECC patients.

Age, gender, and race are recognized as risk factors associated with ECC. Literature reports the median diagnosis age for ECC patients to be 72 years [1], which is higher than the onset age for intrahepatic cholangiocarcinoma. With increasing onset age, the prognosis for ECC patients worsens [11]. Zhao et al. [12] statistically analyzed 1,110 patients with distal bile duct cancer (DBDC) included in the SEER database and found that gender was an independent prognostic factor, with women having a lower incidence than men and a better prognosis compared to men. One study showed that women with ECC tended to have longer survival rates compared to men [13]. Consistent with these findings, in this study, elderly patients aged ≥ 60 accounted for a larger proportion compared to younger patients (79.9% vs. 20.1%), and the OS for women was also better than for men. Race has also been associated with the incidence of ECC, with some studies showing a higher incidence in Asian races than in whites and a lower incidence in blacks than in whites [1]. Our study group included only patients with metastatic (M1) ECC, the majority of whom were white (75.1%), followed by blacks (10%) and other races (15%), and therefore differed from the results of these studies. We hypothesize that ethnic minorities have different access to health care than other races, which may be one reason why the results of this study differ from those of other studies.

Our research findings indicate a notable decrease in OS rates for male ECC patients with lung metastasis, showing a median survival of 5 months for patients without lung metastasis and 2 months for those with lung metastasis (*p* = 0.016). Surprisingly, among women, the survival of patients is not greatly impacted by the existence of lung metastasis, with a median survival of 4 months for those with lung metastasis and 5 months for those without (*p* = 0.19). Studies have shown that female patients are usually diagnosed earlier, have lower levels of total and direct bilirubin at diagnosis, and have relatively better liver function [13], thereby increasing the likelihood of receiving chemotherapy, factors that may influence disease progression and response to treatment. Moreover, the frequent occurrence and spread of ECC, particularly to the lungs, significantly impact the overall survival rate of patients [14]. Huang et al. [15] included 1922 patients from the SEER database and found that lung metastasis was an independent risk factor for ECC by multifactorial COX regression modeling, and patients with lung metastasis had significantly worse OS. These results are consistent with our findings. Wang et al. [6] reported that the lung is the most common site of combined dual‐organ metastases and that patients with the presence of lung metastases may be at a higher risk of liver, bone, brain, and lymph node metastases, which is important for screening and organ‐targeted therapy in patients with ECC. Therefore, lung metastasis should be considered during the diagnosis of ECC, and CT screening of the lungs upon patient admission is particularly important. This may help clinicians better assess and select appropriate treatments, and even perform lung biopsy for confirmation. Although lung metastasis is generally an uncommon manifestation, current research suggests that considering these factors is essential when developing individualized treatment plans.

Chemotherapy was linked to better OS in patients with lung metastasis in our group. Despite the use of OS as the primary endpoint for ECC patients in this study, a meta‐analysis of the literature revealed no significant disparities in cancer‐specific survival (CSS) and OS among various types of BTC [16]. Zhang et al. [17] also reported significant improvements in both OS and CSS for ECC patients after chemotherapy.

This paper has some limitations; the SEER database has some limitations on the sample, such as specific chemotherapy regimens that were not recorded. Also, the date of the data collected in this study is up to 2021, whereas immunotherapy was not recommended until early 2023, which means that we cannot know how the addition of immunotherapy would affect patients with metastatic ECC. In addition, while chemotherapy has been associated with improved survival, the lack of information in the SEER database about patient comorbidities, among other things, that affect a patient's eligibility for chemotherapy may affect survival outcomes. Although chemotherapy patients are often required to be in good health, which may mitigate this confounding effect, future analyses need to consider these comorbidities, and further randomized controlled trials are needed to corroborate ideas.

In conclusion, our study suggests that the presence of lung metastases is associated with reduced survival in patients with ECC. Although chemotherapy significantly prolonged the survival of the patients involved, the overall survival rate of ECC patients with lung metastases remained low. Therefore, they should be further stratified and analyzed in more clinical trials in the future.

## 5 Conclusions

Studies have concluded that lung metastasis is an important prognostic factor in ECC and is associated with poorer survival, especially in male patients. Therefore, preventive measures and effective control of pulmonary metastases (e.g., chemotherapy), especially in male patients, may improve survival in patients with ECC.

## Author Contributions

**Chao Zhang:** conceptualization (equal), formal analysis (equal), methodology (equal), writing – original draft (equal). **Shun Tu:** data curation (equal), software (equal), validation (equal), writing – original draft (equal). **Yanting Liao:** formal analysis (equal), investigation (equal), writing – original draft (equal). **Yaqiang Shu:** validation (equal), visualization (equal), writing – original draft (equal). **Muyu Fu:** data curation (equal), software (equal), writing – original draft (equal). **Jiayue Li:** data curation (equal), formal analysis (equal), writing – original draft (equal). **Xiaohua Lei:** conceptualization (equal), funding acquisition (equal), resources (equal), supervision (equal), writing – review and editing (equal).

## Disclosure

All claims expressed in this article are solely those of the authors and do not necessarily represent those of their affiliated organizations, or those of the publisher, the editors, and the reviewers. Any product that may be evaluated in this article or claim that may be made by its manufacturer is not guaranteed or endorsed by the publisher.

## Ethics Statement

The authors have nothing to report.

## Consent

The authors have nothing to report.

## Conflicts of Interest

The authors declare no conflicts of interest.

## Data Availability

Data used in this study are de‐identified and publicly available. This data can be found here: https://seer.cancer.gov/.
